# Genetic Epidemiology of Glioblastoma Multiforme: Confirmatory and New Findings from Analyses of Human Leukocyte Antigen Alleles and Motifs

**DOI:** 10.1371/journal.pone.0007157

**Published:** 2009-09-23

**Authors:** Wei Song, Avima M. Ruder, Liangyuan Hu, Yufeng Li, Rong Ni, Wenshuo Shao, Richard A. Kaslow, MaryAnn Butler, Jianming Tang

**Affiliations:** 1 Department of Epidemiology, University of Alabama at Birmingham, Birmingham, Alabama, United States of America; 2 National Institute for Occupational Safety and Health, Centers for Disease Control and Prevention, Cincinnati, Ohio, United States of America; 3 Department of Medicine, University of Alabama at Birmingham, Birmingham, Alabama, United States of America; Cedars-Sinai Medical Center and University of California Los Angeles, United States of America

## Abstract

**Background:**

Human leukocyte antigen (HLA) class I genes mediate cytotoxic T-lymphocyte responses and natural killer cell function. In a previous study, several *HLA-B* and *HLA-C* alleles and haplotypes were positively or negatively associated with the occurrence and prognosis of glioblastoma multiforme (GBM).

**Methodology/Principal Findings:**

As an extension of the Upper Midwest Health Study, we have performed HLA genotyping for 149 GBM patients and 149 healthy control subjects from a non-metropolitan population consisting almost exclusively of European Americans. Conditional logistic regression models did not reproduce the association of HLA-B*07 or the B*07-Cw*07 haplotype with GBM. Nonetheless, HLA-A*32, which has previously been shown to predispose GBM patients to a favorable prognosis, was negatively associated with occurrence of GBM (odds ratio = 0.41, *p* = 0.04 by univariate analysis). Other alleles (A*29, A*30, A*31 and A*33) within the A19 serology group to which A*32 belongs showed inconsistent trends. Sequencing-based *HLA-A* genotyping established that A*3201 was the single A*32 allele underlying the observed association. Additional evaluation of *HLA-A* promoter and exon 1 sequences did not detect any unexpected single nucleotide polymorphisms that could suggest differential allelic expression. Further analyses restricted to female GBM cases and controls revealed a second association with a specific *HLA-B* sequence motif corresponding to Bw4-80Ile (odds ratio = 2.71, *p* = 0.02).

**Conclusions/Significance:**

HLA-A allelic product encoded by A*3201 is likely to be functionally important to GBM. The novel, sex-specific association will require further confirmation in other representative study populations.

## Introduction

Glioblastoma multiforme (GBM, also known as Grade IV glioma) is the most common and most severe form of primary brain cancer, with well-documented molecular heterogeneity and rapid fatality [1]–[7]. In the United States, age-adjusted GBM rates are 2.5 times higher in European Americans than in African Americans and 60% higher in men than in women [1], [2], [8], [9]. With varying degrees of certainty, additional factors associated with GBM range from occupational and dietary hazards to reproductive hormones, infectious agents, and variations in genes that regulate DNA repair, carcinogen metabolism, cell cycle, or inflammatory and immune responses [10]. Overall, genetic, developmental and environmental factors are all likely contributors to the etiology and pathogenesis of GBM.

Genes encoding the highly polymorphic human leukocyte antigens (HLA) are known to mediate inflammatory diseases, immune disorders, infectious diseases, and human malignancies [11], [12]. These and other clustered genes form the major histocompatibility complex (MHC) on the short arm of chromosome 6 (6p21.3) and most have dual roles in innate and adaptive immune responses. Multiple HLA alleles and haplotypes have been associated with GBM [13]–[16] as well as other malignancies, including nasopharyngeal carcinoma [17]–[19] and cervical cancer [20]–[22]. Some of these reported associations have been partially replicated and/or validated in studies of immune function [23]–[25], while most appear to be population- or study-specific findings with largely dubious pathogenetic implications.

In previous work based on 155 GBM patients and 157 healthy control subjects recruited from the San Francisco Bay area, several HLA factors have been associated with GBM occurrence and its prognosis [16]. Our follow-up study in a different population now provides further evidence that at least one *HLA-A* allele known as A*3201 may well be a favorable allele that deserves further investigation.

## Results

### Overall characteristics of the study population

Nested within the Upper Midwest Health Study (UMHS) [26], [27], 149 GBM patients and 149 healthy control subjects (**Table 1**) were selected based on 1∶1 matching for four criteria, i.e., ethnicity, sex, age and county of residence. As a result, patients and controls were highly comparable in ethnic background, age and sex ratio, although four African American (AA) patients had to be paired with European Americans (EA) controls. Body mass index, which was not used as a selection criterion, was also quite similar between GBM patients and healthy control subjects (*p* = 0.469). These characteristics formed the basis for conditional logistic regression analyses of HLA genotypes in the paired GBM patients and controls.

**Table 1:** Characteristics of glioblastoma multiforme (GBM) patients and healthy control subjects selected from the Upper Midwest Health Study.

	GBM patients	Healthy controls	*p*
Number of subjects	149	149	―
Sex ratio: F/M	61/88 (0.69)	61/88 (0.69)	―
Age (year)			
Mean±SE	51.7±1.1	52.6±1.1	0.557
Range	18–76	21–77	―
Ethnicity: EA/other	145/4	149/0	0.122
Body mass index (kg/m^2^)			
Mean±SD	25.6±4.1	26.0±4.2	0.469
Range	18.3–39.2	18.8–41.7	―

*P* values≥0.75 are omitted (–); EA = European American, F = female, M = male, SD = standard deviation, SE = standard error of the mean.

### Analyses of HLA alleles and haplotypes

PCR-based genotyping for three HLA class I genes (*HLA-A*, -*B* and –*C*) and one class II locus (*HLA*-*DRB1*) was successful for all 149 case-control pairs. Within each locus, the global distribution of common alleles (frequency≥0.01 in any given population) was similar (*p*>0.50) between the UMHS population and another population studied earlier (**Table 2**), as were the patterns of pairwise linkage disequilibrium (LD) among alleles from different loci (data not shown). Minor differences were noted for a few individual alleles, including A*32, B*14, B*55, and Cw*08 (*p*≤0.025 by univariate Chi-square or Fisher exact tests).

**Table 2:** Distribution of relatively common *HLA-A*, -*B*, -*C*, and -*DRB1* variants in similar case-control populations studied here (this study, *N* = 298) and elsewhere (*N* = 312).

*HLA-A* allele frequency			*HLA-B* allele frequency			*HLA-C* allele frequency			*HLA-DRB1* allele frequency		
Alleles	Elsewhere	This study	Alleles	Elsewhere	This study	Alleles	Elsewhere	This study	Alleles	Elsewhere	This study
A*01	0.172	0.171	B*07	0.130	0.128	Cw*01	0.032	0.022	*01	0.104	0.106
A*02	0.289	0.310	B*08	0.096	0.111	Cw*02	0.045	0.052	*03	0.131	0.114
A*03	0.111	0.141	B*13	0.019	0.032	Cw*03	0.131	0.153	*04	0.149	0.161
A*11	0.069	0.057	B*14	0.042	0.018	Cw*04	0.112	0.111	*07	0.117	0.134
A*23	0.034	0.020	B*15	0.074	0.081	Cw*05	0.080	0.086	*08	0.035	0.040
A*24	0.083	0.079	B*18	0.055	0.052	Cw*06	0.088	0.109	*09	0.010	0.008
A*25	0.022	0.025	B*27	0.037	0.042	Cw*07	0.293	0.310	*10	0.013	0.012
A*26	0.034	0.022	B*35	0.093	0.087	Cw*08	0.050	0.017	*11	0.103	0.092
A*29	0.039	0.025	B*37	0.018	0.027	Cw*12	0.066	0.070	*12	0.018	0.018
A*30	0.030	0.022	B*38	0.018	0.013	Cw*14	0.016	0.008	*13	0.141	0.121
A*31	0.018	0.017	B*40	0.067	0.082	Cw*15	0.040	0.022	*14	0.026	0.027
A*32	0.026	0.050	B*44	0.130	0.153	Cw*16	0.034	0.030	*15	0.135	0.149
A*33	0.018	0.005	B*49	0.021	0.008	Cw*17	0.011	0.010	*16	0.019	0.017
A*68	0.050	0.045	B*51	0.058	0.042	Others	0.002	0			
Others	0.008	0.011	B*52	0.016	0.020						
			B*55	0.037	0.010						
			B*57	0.027	0.042						
			Others	0.064	0.049						

Previously studied population consists of European Americans from the San Francisco Bay area [16]. Rare alleles at each locus are grouped together (others), with number of chromosomes (*2N*) used as the denominator in all tabulations. Between study populations, statistically significant differences (*p*≤0.025) are seen with A*32, B*14, B*55, and Cw*08.

GBM patients and healthy controls were compared for 11 *HLA-A*, 14 *HLA-B*, 10 *HLA-C* and 11 *HLA-DRB1* alleles in a total of 46 univariate models. Three variants, i.e., A*32 (*n* = 29), B*14 (*n* = 11), and B*40 (*n* = 46), were found to be over-represented among the healthy control group (*p* = 0.030 to 0.054) (**Table 3**), while Cw*05 (*n* = 50) was more common in GBM patients (22.2%) than in controls (11.4%) (odds ratio = 2.21, 95% confidence interval = 1.17–4.17). In contrast, no other alleles highlighted in earlier studies [13]–[16] showed any appreciable trend for positive or negative associations with the occurrence of GBM. Further analyses of local and extended haplotypes in this study population also failed to detect any notable relationships.

**Table 3:** Univariate analyses of HLA variants showing clear trend for association with occurrence of glioblastoma multiforme (GBM) in the Upper Midwest Health Study.

HLA variant	In GBM patients	In healthy controls	*p*	OR	95% CI
A*32	9 (6.0)	20 (13.4)	0.040	0.41	0.18–0.94
B*14	2 (1.3)	9 (6.0)	0.054	0.21	0.05–0.99
B*40	16 (10.7)	30 (20.1)	0.030	0.48	0.25–0.92
Cw*05	33 (22.2)	17 (11.4)	0.014	2.21	1.17–4.17

Numbers below each group correspond to n (%) and *p* values are based on maximum likelihood Chi-square test or Fisher exact test (for B*14 only) for 149 GBM patients and 149 healthy controls. OR = odds ratio, CI = confidence interval.

Multivariable analyses dismissed B*14 and B*40 as independent factors (adjusted *p* = 0.070 and 0.118, respectively). In the reduced multivariable model, A*32 retained its negative association with GBM (adjusted OR = 0.39, 95% CI = 0.16–0.91, and *p* = 0.024), with Cw*05 being the only variant showing positive association (adjusted OR = 2.48, 95% CI = 1.24–4.97, and *p* = 0.011). Sequencing of *HLA-A* exons 2 to 4 revealed that A*3201 was the only A*32 allele in the study population. Similar sequencing strategy confirmed that Cw*0501 was the only allele representing Cw*05.

### Insights gained from *HLA-A* promoter and exon 1 sequences

**Figure 1:**
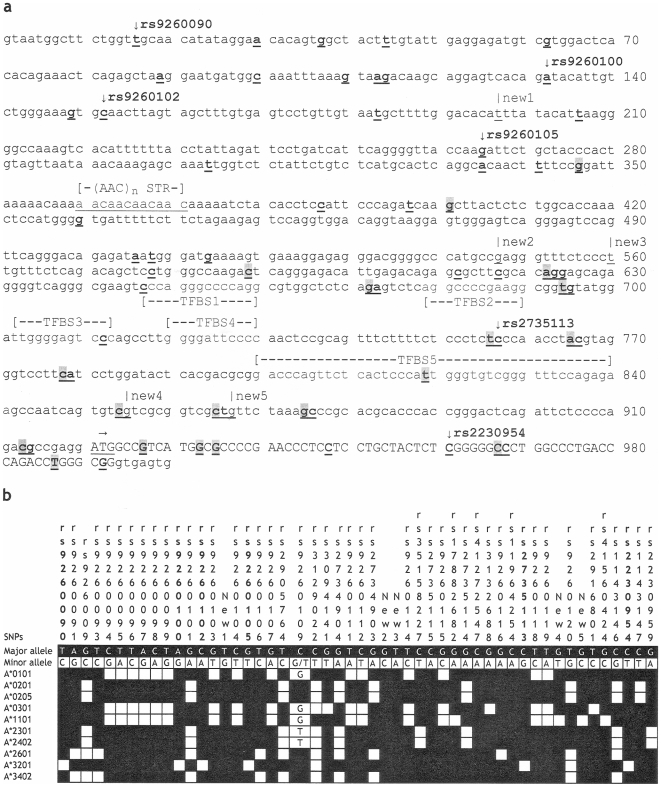
DNA polymorphisms within *HLA-A* promoter and exon 1 sequences. A 1000-bp region (Panel a) has been sequenced for select population samples. Upper case letters are cDNA sequences (part of the open reading frame); the translation start codon (ATG) is indicated by a horizontal arrow. STR denotes a short tandem repeat sequence that has either three or four AAC repeats. Five transcription factor-bindings sites (TFBS) are also indicated. Within this fragment, 69 single nucleotide polymorphisms (SNPs) (bold and underlined) have already been reported in the literature. Those (*n* = 19) not confirmed in this work are shaded grey. The five novel SNPs are designated as “New” (underlined nucleotides below vertical lines). The SNPs unique to A*3201 are marked by vertical arrows before their respective reference sequence (rs) numbers (from dbSNP database, version 126). In panel b, 53 informative SNPs (minor allele frequency ≥0.02) are linked to 11 HLA alleles found in homozygous state.

Selective sequencing of a 1000-bp fragment of *HLA-A* detected 51 SNPs at frequency ≥0.02 (**Figure 1a**); five had no known reference sequence (rs) number in the dbSNP database (version 126). Strong pairwise LD among some SNPs produced four apparent haplotype blocks, each having 3–23 SNPs (**Figure S1**). Regardless of DNA source (GBM patients or control subjects), A*3201 had six unique SNPs (**Figure 1b**), one of which (rs2230954) is nonsynonymous (Ser to Leu substitution) in the first exon. The other five (rs9260090, rs9260100, rs9260102, rs9260105 and rs2735113) are around the core promoter sequences, without any known or predictable functional attributes. DNA sequencing also allowed the assembly of homozygous sequences for 10 common *HLA-A* alleles (**Figure 1b**). A neighbor-joining tree (**Figure S2**) revealed topologies that were identical to known taxonomic hierarchy for their entire open reading frames [28].

### Analyses of HLA class I sequence motifs

*HLA-A*, *-B*, and *–C* sequence motifs were defined by 43, 66, and 28 specific probes in the respective SSO assays. Most (81% to 89%) of them had enough inter-individual variations to be suitable for comparative analyses. The presence of the *HLA-A* motif defined by SSO probe 34 was negatively associated with GBM (OR = 0.50, 95% CI = 0.27–0.91, and *p* = 0.024) (**Table 4**). Common allele groups known to carry this motif include A*23, A*29, A*31, A*32 and A*33 (A*74 was not detected). With the exception of A*23, these allele groups all belong to the A19 serology group [29]. However, individuals whose DNA bound to SSO probe 34 in the absence of A*32 (A*3201) were no less likely to be cases than controls (OR = 0.59, 95% CI = 0.27–1.29, and *p* = 0.183), because modest trends seen with A*23 (8 patients versus 4 controls), A*31 (6 versus 4) and A*33 (3 versus 0) was reversed by A*29 (6 versus 9). Likewise, a common *HLA-B* motif defined by SSO probe 62 had a positive association (OR = 1.87, 95% CI = 1.13–3.10, and *p* = 0.015). Multiple *HLA-B* alleles (e.g., B*14, B*15, B*35, B*44, B*45, B*49, B*50, B*51, B*53, and B*57) are known to have this motif, but none of these alleles were individually associated with GBM (*p*>0.15 in all tests).

**Table 4:** Individual HLA class I sequence motifs associated with the occurrence of glioblasotma multiforme (GBM) in the Upper Midwest Health Study population (*N* = 298 subjects) or in the female subset (61 GBM patients and 61 healthy controls).

HLA motif	In GBM cases	In healthy controls	*p*	OR^c^	95% CI^c^
In all subjects					
*HLA-A*, probe 34	26 (17.5)	42 (28.2)	0.024	0.50	0.27–0.91
*HLA-B*, probe 62	114 (76.5)	94 (63.1)	0.015	1.87	1.13–3.10
In females only					
*HLA-A*, probe 42	24 (39.3)	37 (60.7)	0.017	0.35	0.15–0.83
*HLA-B*, probe 30	21 (34.4)	9 (14.8)	0.024	2.71	1.14–6.46
*HLA-B*, probe 34	21 (34.4)	9 (14.8)	0.024	2.71	1.14–6.46

As described in the text, HLA motifs are defined by individual sequence-specific oligonucleotide (SSO) probes, including *HLA-B* probes 30 and 34 that are in exclusive linkage disequilibrium (*r* ^2^ = 1.0). The *p* values correspond to maximum likelihood estimates. OR = odds ratio, CI = confidence interval.

Despite reduced statistical power, separate analyses of males and females revealed three more sequence motifs that appeared to be associated with GBM in females only (**Table 4**). The *HLA-A* sequence motif defined by SSO probe 42 showed a negative association (OR = 0.35, 95% CI = 0.15–0.83, and *p* = 0.017). Relatively common alleles with this motif include A*01, A*11, A*25, and A*26. *HLA-B* probes 30 and 34 had identical positive association (OR = 2.71, 95% CI = 1.14–6.46, and *p* = 0.024) because they were in exclusive (100%) LD (*r* ^2^ = 1.0). Probe 34 corresponds to a subset of alleles having the Bw4 serological specificity, including B*15 (B*1510 and B*1517), B*39, B*49, B*51, B*53, and B*57. Multivariable analyses supported the independent associations of *HLA-A* and *HLA-B* motifs captured by probes 42 and 34, respectively (adjusted *p*≤0.017) (**Table 4**).

Among the specific sequence motifs of interest, *HLA-A* probes 34 and 42 corresponds to four codons, 149-GCG, 151-CGT, 152-GTG and 153-GCG, and three codons, 161-SAG (where S is either G or C), 163-CGG and 165-GTG, respectively. *HLA-B* probe 30 detects four codons (75-CGA, 77-AAC, 78-CTG and 80-ATC), which have partial overlap with three codons (80-ATC, 81-GCG and 83-CGC) detected by *HLA-B* probe 34. Thus, *HLA-B* probes 30 and 34 share specificity for *HLA-B* codon 80-ATC (AUC in RNA, for Ile). Four more codons defined by the *HLA-B* probe 62 are 161-GAG (for Glu), 162-GGC (Gly), 163-CTG (Leu) and 164-TGC (Cys).

### Genotypes of two SNPs with broad implications for human malignancies

Consistent with results from the CEPH DNA samples analyzed by the International HapMap Project, SNPs rs401681 and rs2736098 in our study population had the minor allele as T and A, respectively. The frequency of rs401681[T] was 0.409 in healthy controls versus 0.440 in GBM cases (*p*>0.65). The rs401681[C] allele has been positively associated with multiple cancers (OR ∼1.2) but negatively associated with melanoma (OR = 0.88) [30]. Here, rs401681[C] was slightly less frequent in GBM cases than healthy controls (OR = 0.88 in test of allele frequency). For SNP rs2736098, the frequency of its minor allele A was 0.338 in healthy controls versus 0.288 in GBM cases (*p*>0.35), in contrast with its positive association with other cancers [30]. Overall, none of the differences in SNP alleles and genotypes (diplotypes) was close to statistical significance.

## Discussion

In several ways, our study of GBM patients and healthy controls from the Upper Midwest Health Study (UMHS) refined and extended findings based on another cohort from the San Francisco Bay area [16]. First, most HLA factors (e.g., B*07, B*13, and Cw*01) revealed by the previous study could not be confirmed here, so their role in the origins of GBM, if any, is unlikely to be generalizable. Second, HLA-A*32 (A*3201) was the only allele that was favorable in both the San Francisco population (prolonged survival) and the Midwest population (protection from disease). Third, specific motifs in the *HLA-A* and *HLA-B* open reading frames appeared to be prominent factors in the Midwest cohort, especially in females. Statistically, age was the most significant difference (*p*<0.0001) between the San Francisco population (mean±standard deviation = 58±12) and the Midwest population (52±13), which might have contributed to inconsistent findings from these cohorts. Environmental factors, including those related to farming [26], [31], could further distinguish the Midwest cohort from the San Francisco cohort. Minor genetic heterogeneity can also offer some alternative explanations, because the frequencies of several *HLA-B* and *HLA-C* alleles differed between the two study populations (**Table 2**). Overall, discordant observations were apparent between the two cohorts despite their close similarity in ethnic background and sample size (statistical power), suggesting that other aspects of study design and population characteristics can be critical to epidemiological analyses.

Aside from the question about relative impact of specific HLA alleles or motifs on GBM in European Americans, our study here and previous work [16] both indicated that the association signals primarily came from the HLA class I region, which, if real, would imply the involvement of cytotoxic T-lymphocyte (CTL) and/or natural killer (NK) cell responses. In that regard, the Bw4 sequence motif (Bw4-80Ile, defined by *HLA-B* probe 34) associated with increased risk for GBM in females is of particular interest, due to its direct role in NK cell activities. Evaluation of two killer immunoglobulin-like receptor (KIR) genes, *KIR3DS1* and *KIR3DL1*, could shed further light on the Bw4 association because these receptors directly or indirectly interact with the Bw4 motif to activate or inhibit NK cell function [32]–[34]. Meanwhile, analyses presented here and elsewhere [16] did not provide any corroboration of positive findings on *HLA-DRB1* genotypes reported in small cohorts [15], [35]. Therefore, HLA class II alleles that dictate T-helper cell function lacked appreciable impact on GBM.

The importance of HLA class I molecules to cancer immunology has been well recognized in experimental studies [12]. In brain cancer, low expression of classical HLA class I genes (*HLA-A*, *-B*, and *–C*) [36] coupled with up-regulation of nonclassical genes (e.g., *HLA-E* and *HLA-G*) likely contributes to immune escape by tumor cells with various somatic mutations [37]–[39]. On the other hand, a study of long-term survivors of anaplastic astrocytoma, which is closely related to GBM [7], has suggested that protective CTLs can effectively respond to glioma-associated antigens [40]. CTLs have indeed been detected in the peripheral blood of GBM patients [41] and antigenic epitopes derived from the alpha 2 chain of interleukin-13 receptor can be presented by HLA-A*02 (A*0201) and A*24 [42]–[44]. It remains to be seen if HLA-A*3201 is advantageous in presenting oncogenic antigens commonly seen in glioma cells [45]–[49]. Patients of African ancestry can be particularly informative as HLA-A alleles in the A19 serology group are most common in African Americans [50], [51]. Epidemiological study of patients with other major forms of brain cancer (e.g., anaplastic astrocytoma) should also help identify favorable HLA factors, which can lead to critical information about the underlying protective mechanisms.

HLA allelic diversity is earmarked by the dominance of nonsynonymous SNPs in the open reading frames, often as a consequence of balancing selection by a variety of human infectious diseases [52]. Such allelic diversity may be equally advantageous in the battle with cancerous cells that frequently switch antigenic repertoire [53]. Thus, in addition to examining the A*3201 open reading frame using routine HLA typing methods, we also partially surveyed regulatory sequences because allele-specific immune surveillance can further depend on allelic expression profile. Our work did reveal five SNP variants in the *HLA-A* promoter region that are likely specific to the A*3201 allele, but none of these is within known transcription factor-binding sites. Expanded analyses of other non-coding sequences around the *HLA-A* locus may help determine whether regulatory sequences beyond the promoter region can separate favorable from unfavorable or neutral alleles, especially when closely related alleles (e.g., A*3201 and others in the A19 serology group) differ in their impact on disease.

In other brain tumor studies that have dealt with candidate genes outside the HLA system (reviewed in ref. 10), the magnitudes of genetic associations (usually with SNP genotypes) have generally been modest. Further evidence from SNP-based genome-wide association studies has been equally unremarkable (less than 2-fold difference), including the recent implication of two SNPs (rs401681 and rs2736098) consistently but weakly associated with a variety of human malignancies [30], as well as other SNP genotypes detected in genome-wide association studies of glioma [54], [55]. Indeed, our analyses of rs401681 and rs2736098 produced only minimal evidence that allele C of the intronic SNP rs401681 (at the *CLPTM1L* locus) is probably unfavorable in brain cancer.

In summary, case-control studies described here and earlier [16] have yielded clues to potential involvement of HLA class I alleles and motifs in GBM. The findings are still difficult to interpret because none of them can be immediately related to other reports on solid tumors. Of note, HLA-A*3201 (A19 or A32 by serology) is a relatively infrequent allele, with an overall carriage (“phenotype”) frequency less than 10% (allele frequency <0.05) in most populations [29], [50], [56]. Lack of information about this allele is not surprising, because even studies of adequate sample size (i.e., hundreds to thousands of cases and controls) can have limited statistical power if the association is weak or obscured by other factors. Bw4-80Ile, on the other hand, is a common variant; hypothesis about Bw4-80Ile can be readily tested. Large collaborative efforts, as promoted by the Brain Tumor Epidemiology Consortium [10], are expected to expedite confirmatory studies of HLA alleles and motifs in other well-defined cohorts, especially those of diverse ethnic backgrounds as well as wide geographic coverage. Recognition of GBM as a molecularly heterogeneous cancer [4], [7] also calls for the separate analyses of primary and secondary glioblastoma, as the latter is closely related to anaplastic astrocytoma (Grade III glioma) [7].

## Materials and Methods

### Study population

We studied unrelated subjects in the Upper Midwest Health Study [26], [27], which enrolled cancer patients and frequency-matched, population-based controls from non-metropolitan areas in four Midwest states (Iowa, Michigan, Minnesota and Wisconsin). All patients with glioblastoma multiforme (GBM = Grade IV glioma, as classified by the World Health Organization) were included if they were 18 years or older at time of GBM diagnosis and blood sampling. Healthy control subjects drawn from this study had no self-reported cancer of any type and were matched to GBM patients at a 1∶1 ratio by sex, state of residence and at least two of three additional criteria, i.e., age (±3 yr), race/ethnicity (self-identified), and county of residence (or adjacent county). The final study population consisted of 149 pairs of GBM patients and healthy controls (**Table 1**). The original research and this substudy conformed to the US Department of Health and Human Services guidelines for protection of human subjects. All patients and healthy controls provided written informed consent. The procedures for obtaining written informed consent, blood sample, clinical information, data management and data analysis were approved by institutional review board (IRB) at the National Institute for Occupational Safety and Health (NIOSH, Protocol HSRB 94-DSHEFS-08). Work related to this substudy was further approved by IRBs at NIOSH and University of Alabama at Birmingham (Protocol X071005007).

### HLA Genotyping

Genomic DNA samples, prepared from whole blood either using the QIAamp blood kit (QIAGEN Inc., Chatsworth, Calif., USA) or by sodium-perchlorate chloroform extraction [27], were used for molecular typing of three HLA class I genes (*HLA-A*, *HLA-B*, and *HLA-C*), along with the most polymorphic HLA class II gene, *HLA-DRB1*. Genotyping relied on a combination of PCR-based techniques commonly used in population-based studies [57], [58]. Briefly, alleles (4-digit designations) and allele groups (2-digit designations) from the three HLA class I genes were first amplified by locus-specific primer mixes and then classified after automated hybridization to sequence-specific oligonucleotide (SSO) probes (Innogenetics, Alpharetta, Georgia, USA). Ambiguous HLA class I genotypes were resolved by sequencing-based typing (SBT), which covered three exons (2–4) in six sequencing reactions (three forward and three reverse) (Abbott Molecular, Inc., Des Plaines, Illinois, USA). Capillary electrophoresis and allele assignments in SBT were done using the ABI 3130xl DNA Analyzer (Applied Biosystems, Foster City, Calif., USA). *HLA-DRB1* alleles in the HLA class II region were directly resolved by sequencing exon 2 in three reactions (forward, reverse, and codon 86) (Abbott Molecular, Inc.). For quality control purposes, randomly selected samples (*n* = 39, or 13% of the total) were genotyped in duplicate.

### Confirmatory sequencing of *HLA-A* promoter and exon 1 sequences

To enhance the interpretation of findings on HLA-A alleles, a 1000-bp region (**Figure 1a**) not targeted in routine genotyping was sequenced using a commercial, high-throughput platform (Polymorphic DNA Technologies, Alameda, Calif., USA). The fragment has the core promoter [59]–[61] and exon 1 sequences, with >60 single nucleotide polymorphisms (SNPs). Eight PCR primers and eight internal sequencing primers (sequences available from JT upon request) were used for bidirectional sequencing in subjects who carried homozygous genotypes or common alleles of interest. Individual SNP genotypes were analyzed for pairwise linkage disequilibrium (LD) using the HaploView program (http://www.broad.mit.edu/haploview/haploview-downloads). Homozygous sequences were also tested for phylogenetic relationships (**Figure S2**) that could be directly compared with known taxonomic hierarchy for protein-coding sequences (open reading frames) [28].

### Selective genoyping of two SNPs with broad implications for human malignancies

For exploratory analyses, two SNPs (rs401681 and rs2736098) recently associated with multiple human cancers [30] were typed for all GBM cases and healthy controls using pre-designed TaqMan (5′ nuclease) assays (assay-on demand IDs C_1150767_20 and C_26414916_20, respectively) (Applied Biosystems, Foster City, CA). Based on procedures recommended by the manufacturer, the SNP assays were run in 6-µL PCR reactions in 96-well plates, with each reaction having 10 ng total genomic DNA mixed with 2× TaqMan Universal PCR Master Mix (Applied Biosystems). Allelic discrimination relied on end-point fluorescence intensity after 35 cycles of PCR (denaturing at 95°C for 15 sec and annealing/extending at 60°C for 60 sec) in an ABI 7500 FAST system (Applied Biosystems). Each plate had four wells for negative controls (no template DNA added) and 3% of all DNA samples were tested in random duplicates for quality control.

### Statistical analyses

Statistical Analysis Software (SAS), version 9.2 (SAS Institute, Cary, North Carolina, USA) was used for all descriptive statistics and comparative analyses. Serial analytical strategies were similar to those reported in prior work [16], with a starting focus on 2-digit allele groups (often equivalent to serological specificities) and linkage disequilibrium (LD) between HLA factors. Only common variants found in at least 10 individuals (∼3.4% of the study population) were formally tested. In all hypothesis testing, a nominal P value≤0.05 was considered statistically significant. Multivariable and conditional logistic regression models with backward or step-wise selection procedure were used to generate the parsimonious models with all independent factors (adjusted multivariable P value≤0.05). Novel associations were reported as such if the univariate P value was <0.05 in conditional logistic regression models. As homozygosity with any given HLA allele or motif (defined by individual SSO probes) was rare, statistical models only tested dominant effects. Analyses of individual SNP genotypes were modeled for recessive effects (homozygosity or two copies of the minor allele), dominant effects (homo- and heterozygosity combined), and additive effects (0, 1 and 2 copies of the minor allele). Estimates of odds ratio (OR) and 95% confidence interval (CI) were the main summary statistics from these analyses.

## Supporting Information

Figure S1 Patterns of linkage disequilibrium (LD) among informative SNPs within *HLA-A* promoter and exon 1 sequences. Novel SNPs without the official reference sequence (rs) numbers are designated as “New.” Among the 51 SNPs with minor allele frequencies ≥0.02 (Figure 1), one (rs9260109) is excluded from this analysis because of three different alleles (i.e., not dimorphic) at this site. Strong pairwise LD (shown in red) leads to the identification of four haplotype blocks (framed), which consist of 13, 23, 6 and 3 SNPs, respectively.
(0.27 MB DOC)

Figure S2 Neighbor-joining tree illustrating the phylogenetic relationships of *HLA-A* promoter and exon 1 sequences representing 11 alleles found in homozygous state. Two alleles (A*260101 and A*320101) have the full, 6-digit designations. Scale of genetic distance is shown at the bottom.
(0.05 MB DOC)
