# Supplementary figures and images for: Genetic Epidemiology of Glioblastoma Multiforme: Confirmatory and New Findings from Analyses of Human Leukocyte Antigen Alleles and Motifs
Source: PLoS One. 2009 Sep 23;4(9):e7157. doi: 10.1371/journal.pone.0007157 (PMC2742900; doi:10.1371/journal.pone.0007157)

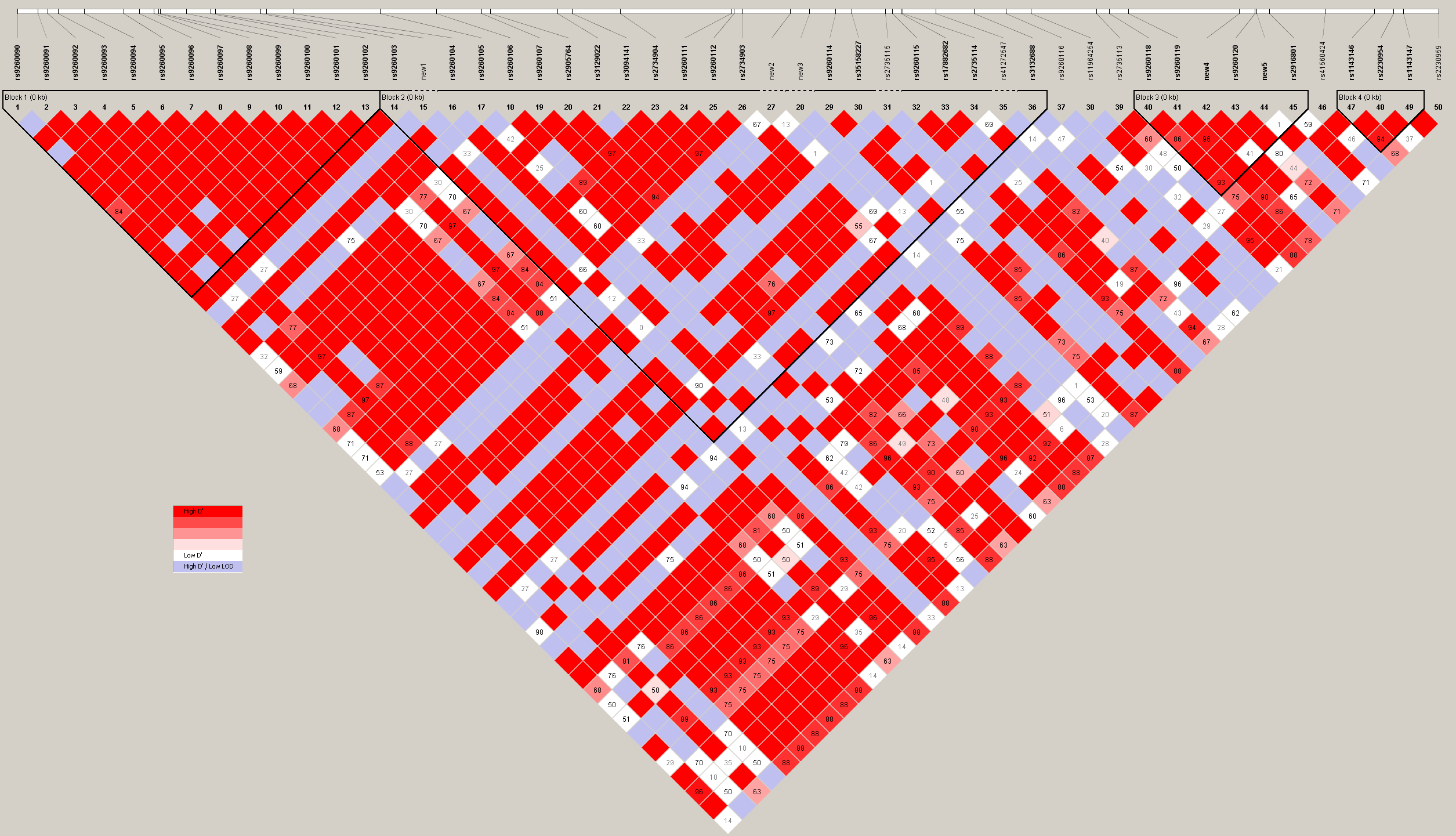

Supplement: Figure S1 — Patterns of linkage disequilibrium (LD) among informative SNPs within HLA-A promoter and exon 1 sequences. Novel SNPs without the official reference sequence (rs) numbers are designated as “New.” Among the 51 SNPs with minor allele frequencies ≥0.02 (Figure 1), one (rs9260109) is excluded from this analysis because of three different alleles (i.e., not dimorphic) at this site. Strong pairwise LD (shown in red) leads to the identification of four haplotype blocks (framed), which consist of 13, 23, 6 and 3 SNPs, respectively. (0.27 MB DOC) [file pone.0007157.s001.doc]

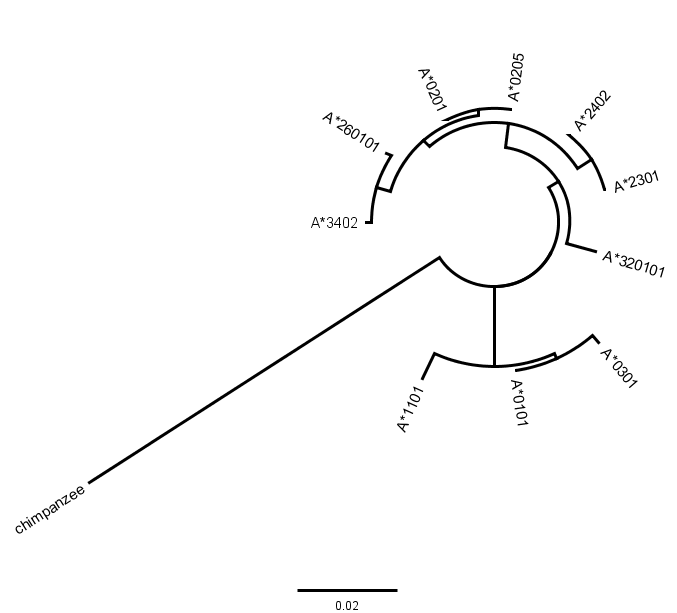

Supplement: Figure S2 — Neighbor-joining tree illustrating the phylogenetic relationships of HLA-A promoter and exon 1 sequences representing 11 alleles found in homozygous state. Two alleles (A*260101 and A*320101) have the full, 6-digit designations. Scale of genetic distance is shown at the bottom. (0.05 MB DOC) [file pone.0007157.s002.doc]
